# Real-world evidence on non-invasive tests and associated cut-offs used to assess fibrosis in routine clinical practice

**DOI:** 10.1016/j.jhepr.2022.100596

**Published:** 2022-09-22

**Authors:** Jeffrey V. Lazarus, Laurent Castera, Henry E. Mark, Alina M. Allen, Leon A. Adams, Quentin M. Anstee, Marco Arrese, Saleh A. Alqahtani, Elisabetta Bugianesi, Massimo Colombo, Kenneth Cusi, Hannes Hagström, Rohit Loomba, Manuel Romero-Gómez, Jörn M. Schattenberg, Maja Thiele, Luca Valenti, Vincent Wai-Sun Wong, Yusuf Yilmaz, Zobair M. Younossi, Sven M. Francque, Emmanuel A. Tsochatzis

**Affiliations:** 1 Barcelona Institute for Global Health (ISGlobal), Hospital Clínic, University of Barcelona, Barcelona, Spain; 2 Faculty of Medicine and Health Sciences, University of Barcelona, Barcelona, Spain; 3 Université de Paris, UMR1149 (CRI), Inserm, Paris, France & Service d’Hépatologie, AP-HP, Hôpital Beaujon, Clichy, France; 4 Division of Gastroenterology and Hepatology, Department of Internal Medicine, Mayo Clinic, Rochester, MN, USA; 5 Department of Hepatology and Liver Transplant Unit, Sir Charles Gairdner Hospital, Medical School, University of Western Australia, Perth, Australia; 6 Translational & Clinical Research Institute, Faculty of Medical Sciences, Newcastle University, Newcastle Upon Tyne, UK; 7 Newcastle NIHR Biomedical Research Centre, Newcastle Upon Tyne Hospitals NHS Trust, Newcastle Upon Tyne, UK; 8 Department of Gastroenterology, Escuela de Medicina, Pontificia Universidad Católica de Chile, Santiago, Chile; 9 Centro de Envejecimiento y Regeneración (CARE), Departamento de Biología Celular y Molecular, Facultad de Ciencias Biologicas, Pontificia Universidad Catolica de Chile, Santiago, Chile; 10 Division of Gastroenterology & Hepatology, Johns Hopkins University, Baltimore, MD, USA; 11 Organ Transplant Center, King Faisal Specialist Hospital & Research Center, Riyadh, Saudi Arabia; 12 Department of Medical Sciences, Division of Gastroenterology and Hepatology, A.O. Città della Salute e della Scienza di Torino, University of Turin, Turin, Italy; 13 Liver Center, IRCCS San Raffaele Hospital, Milan, Italy; 14 Division of Endocrinology, Diabetes and Metabolism, University of Florida, Gainesville, FL, USA; 15 Department of Medicine, Huddinge, Karolinska Institutet, Stockholm, Sweden; 16 Department of Medicine, NAFLD Research Center, La Jolla, CA, USA; 17 Department of Medicine, University of California San Diego, La Jolla, CA, USA; 18 Virgen del Rocio University Hospital, Institute of Biomedicine of Seville (HUVR/CSIC/US), CIBEREHD, University of Seville, Seville, Spain; 19 Metabolic Liver Research Program, I. Department of Medicine, University Medical Centre Mainz, Mainz, Germany; 20 Liver Research Center, Odense University Hospital and University of Southern Denmark, Odense, Denmark; 21 Department of Pathophysiology and Transplantation, Università degli Studi di Milano, Milan, Italy; 22 Precision Medicine, Department of Transfusion Medicine and Hematology, Fondazione IRCCS Ca’ Granda Ospedale Maggiore Policlinico, Milan, Italy; 23 Medical Data Analytics Centre, Department of Medicine and Therapeutics, The Chinese University of Hong Kong, Hong Kong, China; 24 Department of Gastroenterology, School of Medicine, Recep Tayyip Erdoğan University, Rize, Turkey; 25 Liver Research Unit, Institute of Gastroenterology, Marmara University, Istanbul, Turkey; 26 Center for Liver Diseases, Inova Medicine, Falls Church, VA, USA; 27 Department of Gastroenterology Hepatology, University Hospital Antwerp & Translational Sciences in Inflammation and Immunology TWI2N, Faculty of Medicine and Health Sciences, University of Antwerp, Antwerp, Belgium; 28 University College London Institute for Liver and Digestive Health, Royal Free Hospital, London, United Kingdom; 29 Sheila Sherlock Liver Centre, Royal Free Hospital, London, United Kingdom

**Keywords:** Hepatology, NAFLD, NITs, FIB-4, Elastography, NFS, FIB-4, Fibrosis-4, NAFLD, Non-alcoholic fatty liver disease, NFS, NAFLD fibrosis score, NIT, Non-invasive test

## Abstract

**Background & Aims:**

Non-invasive tests (NITs) offer a practical solution for advanced fibrosis identification in non-alcoholic fatty liver disease (NAFLD). Despite increasing implementation, their use is not standardised, which can lead to inconsistent interpretation and risk stratification. We aimed to assess the types of NITs and the corresponding cut-offs used in a range of healthcare settings.

**Methods:**

A survey was distributed to a convenience sample of liver health experts who participated in a global NAFLD consensus statement. Respondents provided information on the NITs used in their clinic with the corresponding cut-offs and those used in established care pathways in their areas.

**Results:**

There were 35 respondents from 24 countries, 89% of whom practised in tertiary level settings. A total of 14 different NITs were used, and each respondent reported using at least one (median = 3). Of the respondents, 80% reported using FIB-4 and liver stiffness by vibration-controlled transient elastography (Fibroscan®), followed by the NAFLD fibrosis score (49%). For FIB-4, 71% of respondents used a low cut-off of <1.3 (range <1.0 to <1.45) and 21% reported using age-specific cut-offs. For Fibroscan®, 21% of respondents used a single liver stiffness cut-off: 8 kPa in 50%, while the rest used 7.2 kPa, 7.8 kPa and 8.7 kPa. Among the 63% of respondents who used lower and upper liver stiffness cut-offs, there were variations in both values (<5 to <10 kPa and >7.5 to >20 kPa, respectively).

**Conclusions:**

The cut-offs used for the same NITs for NAFLD risk stratification vary between clinicians. As cut-offs impact test performance, these findings underscore the heterogeneity in risk-assessment and support the importance of establishing consistent guidelines on the standardised use of NITs in NAFLD management.

**Lay summary:**

Owing to the high prevalence of non-alcoholic fatty liver disease (NAFLD) in the general population it is important to identify those who have more advanced stages of liver fibrosis, so that they can be properly treated. Non-invasive tests (NITs) provide a practical way to assess fibrosis risk in patients. However, we found that the cut-offs used for the same NITs vary between clinicians. As cut-offs impact test performance, these findings highlight the importance of establishing consistent guidelines on the standardised use of NITs to optimise clinical management of NAFLD.

## Highlights

• NITs are a valuable tool to identify patients with NAFLD and fibrosis who require specialist care.
• Lower and upper cut-offs have important implications for test performance and clinical decision making.
• Among 35 survey respondents, 14 different NITs were used, of which FIB-4 and transient elastography were the most common.
• This study is the first to describe the considerable heterogeneity in the NIT cut-offs used by clinicians in different settings.
• Guidelines to standardise NIT cut-offs are needed to improve consistency in risk-stratification in NAFLD.

## Graphical abstract

**Figure:**
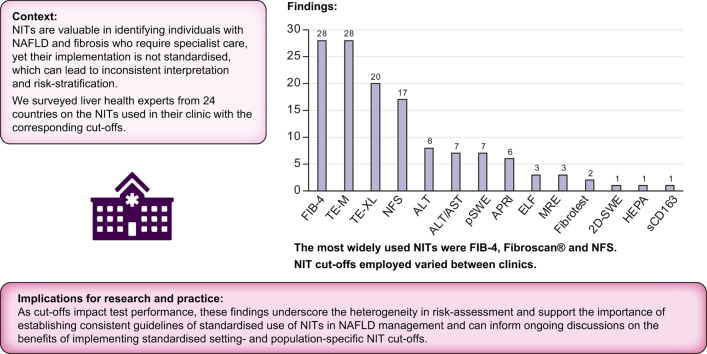

## Introduction

One of the enduring challenges of addressing the burden of non-alcoholic fatty liver disease (NAFLD) is ensuring that individuals with advanced stages of liver fibrosis are identified and provided with appropriate care by liver health specialists.^1^ Non-invasive tests (NITs) provide a practical way to assess fibrosis risk in patients.^2^ NITs fall within two broad categories: 1) serum biomarkers; and 2) liver stiffness measured by ultrasound or magnetic resonance-based elastography techniques.^3^ Currently available NITs are most reliable for ruling out advanced stages of fibrosis (*i.e.*, stages 3-4 on the Non-Alcoholic Steatohepatitis Clinical Research Network and Steatosis-Activity-Fibrosis staging systems).^4^^,^^5^

The cut-offs employed have important implications for the sensitivity and specificity of NITs and the size of the indeterminate range. Generally, a low cut-off will improve the sensitivity and negative predictive value, and is therefore suited for ruling out advanced fibrosis, while a high cut-off will improve the specificity, positive predictive value and ability to rule it in. The most commonly used diagnostic outcome for risk stratification of individuals with NAFLD is advanced fibrosis (stages 3-4) due to its prognostic value. Due to the typically low prevalence of advanced fibrosis among populations with NAFLD, the negative predictive value of NITs is generally high, meaning that individuals with results below the cut-off can temporarily be excluded from further investigations, with a high degree of confidence. This is the goal in primary care, where the aim is to select individuals at risk of progressive liver disease for referral to specialist care, while ensuring that no individuals with the disease are missed. However, the positive predictive value of the tests is typically lower, meaning that single NITs are unable to provide a definitive diagnosis.^6^ In contrast, in secondary and tertiary care, where the focus is on diagnostic confirmation, the higher cut-offs used generate a high specificity and high positive predictive value.^7^

NITs – used as stand-alone tests or in combinations (simultaneous or sequential) – are increasingly used in primary and secondary care to identify individuals for referral to a liver specialist. In some settings, NITs have facilitated the development of formal care pathways that aim to efficiently and effectively link patients to care, especially those with advanced liver disease who require intervention from a hepatologist/liver specialist or multidisciplinary team.^8^ In developing these pathways, decisions need to be made about which cut-offs should be used based on the clinical scenario.

While NITs are becoming more widely utilised as a means of identifying individuals with NAFLD and advanced fibrosis, little is known about the cut-offs being employed in clinical practice. Many reports include cut-offs for a specific study population – often transposing cut-offs identified in other aetiologies of chronic liver disease – leading to a range of published cut-offs. We hypothesise that the NITs used, and the corresponding cut-offs applied in practice, are widely heterogeneous between different healthcare settings and practices. In this brief report, we aim to explore the different NITs and corresponding cut-offs being used in routine clinical practice in a range of healthcare settings.

## Materials and methods

In March 2021, a short survey (see supplementary material) was distributed to a convenience sample of 215 liver health experts who participated in a NAFLD consensus statement process in early 2021;^9^ completed surveys were returned by August 2021. Comprised of three parts, the survey collected information on: the respondents’ clinical setting, including the level of care (*i.e*., primary, secondary, tertiary), and the predominant patient population seen in the clinic; the NITs used in the clinic and those used in formal care pathways in the respondents’ setting; and the cut-offs employed and the existence of national and sub-national risk stratification pathways. We provide a descriptive analysis of the findings, including the variations in NIT cut-offs reported by respondents.

## Results

A total of 35 survey responses were received. Most respondents (31/35; 89%) described their clinic as being in a tertiary level hospital setting, while the rest were from secondary level settings that all managed individuals with confirmed or suspected liver disease. Respondents were from a total of 24 countries. Most respondents were based in Europe (21/35; 60%), followed by East Asia and the Pacific (6/35; 17%). Two clinics were based in each of Latin America and the Caribbean and the Middle East and North Africa (6%) and one each in North America, sub-Saharan Africa, Central Asia and South Asia (3%).

**Fig. 1:**
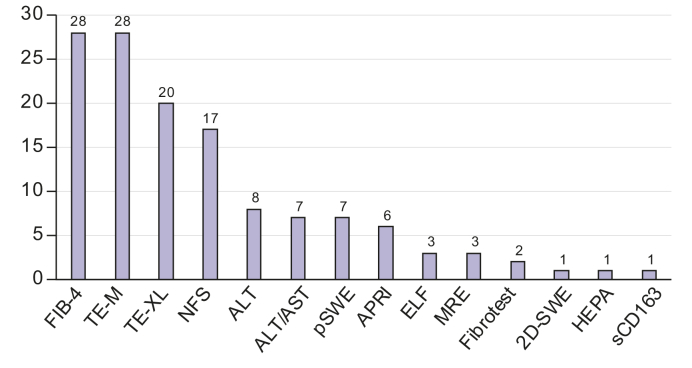
Number of clinics (n = 35) using each type of non-invasive test. 2D-SWE, 2-dimensional shear wave elastography; ALT, alanine aminotransferase; APRI, aspartate aminotransferase-to-platelet ratio index; AST, aspartate aminotransferase; ELF, Enhanced Liver Fibrosis; FIB-4, Fibrosis-4; HEPA, HEPAmet fibrosis score; MRE, magnetic resonance elastography; NFS, NAFLD fibrosis score; pSWE, point shear wave elastography; sCD163, Soluble Cluster of Differentiation 163; TE-M, transient elastography-M; TE-XL, transient elastography-XL.

Across the 35 settings, 14 different NITs were used, with each respondent reporting the use of at least one NIT (Fig. 1) (median = 3; range 1-8). Fibrosis-4 (FIB-4) and transient elastography (Fibroscan®) were the most used, reported by 28 of the 35 respondents (80%), followed by the NAFLD fibrosis score (NFS) (17/35; 49%).

Overall, respondents from 11 countries (11/24; 46%) reported that a national risk stratification pathway exists which outlines NIT cut-offs, while 7 respondents (7/35; 20%) reported that a sub-national risk stratification pathway exists.

For FIB-4, 71% of respondents (20/28) reported a low cut-off of <1.3. The lowest low cut-off used was <1.0, while the highest low cut-off used was <1.45. Six respondents (21%) reported age-specific cut-offs for FIB-4, with five (83%) employing a low cut-off of <1.3 for patients ≤65 and of <2.0 for those >65, as has previously been proposed.^10^ Five respondents (18%) reported the use of a single FIB-4 cut-off, while the rest employed an upper cut-off, with an intermediate range between the upper and lower thresholds. Of these 23, 11 (48%) used an upper cut-off of >2.67 while 9 (39%) used >3.25.

Of the 28 respondents reporting the use of Fibroscan®, six (21%) used a single cut-off; three of these (50%) used 8 kPa as the cut-off, while the remaining used 7.2 kPa, 7.8 kPa and 8.7 kPa. Of the respondents employing an upper and lower cut-off, these ranged from <5 kPa (3/22; 14%) to <10 kPa (3/22; 14%), with the most common lower cut-off of <8.0 kPa being reported by 7 respondents (32%). Upper cut-offs varied from >7.5 kPa to >20 kPa, with 15 kPa being the most used (5/22; 23%).

NFS, the third most used NIT, had the least variation in cut-offs, with all 17 respondents using <1.455 as the low cut-off threshold. One respondent reported a single low cut-off and the remainder used a high cut-off, which ranged from >0.672 to >0.676, with the latter being reported by 10 respondents (59%).

## Discussion

The findings reveal that the cut-offs employed for the same NITs vary between individual practitioners, especially for the high cut-offs, which aim at ruling in advanced fibrosis. The level of variation in cut-offs differs by NIT, with cut-offs varying much less for some tests, such as NFS, than others, including FIB-4 and Fibroscan®. As lower and upper cut-offs have important implications for the sensitivity and specificity of the test, these findings can inform ongoing discussions around the benefits of using standardised cut-offs for specific settings and population groups.

Some of the variation identified may result from the different approaches employed across clinical settings. In some settings, clinicians are using the high negative predictive value of NITs as a means of ruling out advanced disease and, where necessary, these tests are followed by further investigations that can include a liver biopsy. Given the lower positive predictive value of NITs, in settings where clinicians use these to make a definitive diagnosis, they may be required to use a higher upper threshold to increase the certainty of the result. Most respondents in this study employed more than one NIT in their setting, and it is increasingly common that care pathways employ NITs simultaneously or sequentially.^11^^,^^12^ Where care pathways use multiple sequential NITS, a lower cut-off to rule-out advanced fibrosis, used prior to a higher cut-off to rule it in, is justified.^13^ Age can also influence the accuracy of NITs, with low specificity for advanced fibrosis in those <35 and low sensitivity in those >65, leading to calls for age-adjusted cut-offs, including for NFS and FIB-4.^10^ In our small study sample, just over one in five clinics used age-adjusted cut-offs for FIB-4.

The liver health field needs to consider the reasons for the variation in NIT cut-offs and the clinical and public health implications that this entails. Focus should be on understanding where the variation arises because of new data and evidence, such as specific cut-offs in different population groups, and where it is the result of a lack of clear, uniform guidance and care pathways, as well as heterogeneity in available biomarkers. Qualitative research approaches would help to elucidate some of these reasons, including interviews with clinicians to understand the rationale behind their use of a particular NIT and cut-off.

NITs are a valuable tool to identify those with NAFLD who require specialist care. These findings show that the cut-offs being used in routine practice vary widely between the 35 respondents. There is a gap in the current literature on the implications of this variation and an urgent need for research to help guide efforts to better understand the implications of different cut-offs on patient outcomes and on health system resourcing. These discussions need to be integrated into broader discussions on advancing more efficient, patient-centred models of care for people living with NAFLD.^8^ Regional and national liver disease associations and other norm setting bodies have a critical role to play in collating and analysing the latest data and incorporating these into clinical practice guidelines.^14^^,^^15^ Respondents from 11 countries indicated that a national risk stratification pathway exists, yet for five of these counties respondents indicated that a pathway did not exist. This points to a definitional issue around what constitutes a national pathway, and the need for a clearer definition of this.

Our study has several strengths and limitations. To our knowledge, this is the first study to present the variation of cut-offs among NITs used in routine clinical practice. These heterogeneous pilot data highlight the need for larger and more detailed studies of this kind, which should also include the corresponding actions based on the test results. Assessment of the current practice landscape is the first step toward standardisation of cut-offs. Our data come from an opportunistic sample of clinicians and researchers engaged in a previous study,^9^ and while there were primary care clinicians in this sample, we received no response from this setting. While the small number of respondents is a limitation, this study does establish that there is a lack of uniform use of NIT cut-off values. Future studies of this kind should be larger and aim to include respondents from primary care, as that is a critical first-line setting for identifying advanced liver disease.^16^ The variability in NIT type and cut-offs used in primary care may be even higher than the one seen among hepatology specialists, given lesser familiarity with the intricacies of biomarkers and the lack of standardisation by guidance documents.^17^

These findings demonstrate that cut-offs used for the same NITs vary between clinicians. As lower and upper cut-offs have important implications for the sensitivity and specificity of the test, *i.e*. ruling advanced fibrosis in or out, these findings can inform ongoing discussions on the benefits of implementing standardised setting- and population-specific cut-offs, and the revision of current testing guidelines.

## Financial support

This study did not receive any funding.

## Authors’ contributions

JVL conceived of the study with input from HEM. HEM and JVL led the data collection and drafted the first iteration of the manuscript. All authors reviewed the full draft of the article, subsequent revisions and approved the final version for submission.

## Data availability statement

Data are available for fair use from the corresponding author upon request.

## Conflict of interest

JVL acknowledges grants and speaker fees from AbbVie, Gilead Sciences and MSD and speaker fees from Genfit, Intercept, Jannsen and ViiV, outside of the submitted work. LC has participated in advisory boards for Alexion, Echosens, MSD, Novo Nordisk, Pfizer and Sagimet and has received lecture fees from Echosens and Novo Nordisk, outside of the submitted work. AMA has received research support from Novo Nordisk, Pfizer, Target Pharma and is a consultant for Novo Nordisk and Pfizer, outside of the submitted work. LAA reports being on the advisory board for Pfizer, Roche Diagnostics and Novartis, outside of the submitted work. QMA reports Research Grant Funding: AbbVie, AstraZeneca, Boehringer Ingelheim, Glympse Bio, Intercept, Novartis, Pfizer. Consultancy on behalf of Newcastle University: Alimentiv, Akero, AstraZeneca, Axcella, 89Bio, Boehringer Ingelheim, Bristol Myers Squibb, Galmed, Genfit, Genentech, Gilead, GlaxoSmithKline, Hanmi, HistoIndex, Intercept, Inventiva, Ionis, IQVIA, Janssen, Madrigal, Medpace, Merck, NGMBio, Novartis, Novo Nordisk, PathAI, Pfizer, Poxel, Resolution Therapeutics, Roche, Ridgeline Therapeutics, RTI, Shionogi, Terns. Speaker: Fishawack, Integritas Communications, Kenes, Novo Nordisk, Madrigal, Medscape, Springer Healthcare. Royalties: Elsevier Ltd. All has been outside of the submitted work. MA has received grants from the Chilean Government: Fondo Nacional de Desarrollo Científico y Tecnológico (FONDECYT 1191145) and the Comisión Nacional de Investigación Científica y Tecnológica (CONICYT, AFB170005, CARE Chile UC). All has been outside of the submitted work. EB served as a consultant or advisory board member for Boehringer Ingelheim, Gilead Sciences, Intercept, Inventiva, Merck, Novo Nordisk, Pfizer, ProSciento and a speaker for Gilead Sciences, MSD and Novo Nordisk, outside of the submitted work. MC has been on the advisory board for Target HCC, Exelixis, Galapagos and Gilead, outside of the submitted work. KC has received research support towards the University of Florida as principal investigator from Echosens, Inventiva, Novo Nordisk, Poxel, Labcorp and Zydus and is a consultant for Arrowhead, AstraZeneca, 89Bio, BMS, Lilly, Madrigal, Novo Nordisk, Quest, Sagimet, Sonic Incytes and Terns, outside of the submitted work. HH’s institution has received research grants from AstraZeneca, Echosens, Gilead, Intercept, MSD and Pfizer, outside of the submitted work. RL serves as a consultant to Aardvark Therapeutics, Altimmune, Anylam/Regeneron, Amgen, Arrowhead Pharmaceuticals, AstraZeneca, Bristol-Myer Squibb, CohBar, Eli Lilly, Galmed, Gilead, Glympse bio, Hightide, Inipharma, Intercept, Inventiva, Ionis, Janssen Inc., Madrigal, Metacrine, Inc., NGM Biopharmaceuticals, Novartis, Novo Nordisk, Merck, Pfizer, Sagimet, Theratechnologies, 89 bio, Terns Pharmaceuticals and Viking Therapeutics. In addition, his institutions received research grants from Arrowhead Pharmaceuticals, Astrazeneca, Boehringer-Ingelheim, Bristol-Myers Squibb, Eli Lilly, Galectin Therapeutics, Galmed Pharmaceuticals, Gilead, Hanmi, Intercept, Inventiva, Ionis, Janssen, Madrigal Pharmaceuticals, Merck, NGM Biopharmaceuticals, Novo Nordisk, Pfizer, Sonic Incytes and Terns Pharmaceuticals. He is co-founder of LipoNexus Inc. All has been outside of the submitted work. MR-G has received grants from Siemens, Gilead and Intercept. He has also served as a consultant for Abbvie, Axcella, Alpha-sigma, BMS, Allergan, Boehringer-Ingelheim, Astra-Zeneca, Gilead, Inventia, Rubió, Kaleido, Siemens, Novo-Nordisk, Shionogi, Pfizer, Sobi, Zydus, Prosciento, Shionogi, MSD and Sobi. In addition he has been lecturer in Inventia, Rubió, Sobi, Novo-Nordisk and Shionogi. He has participated in an advisory board for Galmed and has received support from AbbVie and Gilead to attend meetings. All has been outside of the submitted work. JMS reports consultant activities for Apollo Endosurgery, Albireo Pharma Inc, Bayer, BMS, Boehringer Ingelheim, Echosens, Gilead Sciences, GSK, Intercept Pharmaceuticals, Ipsen, Inventiva Pharma, Julius Clinical, Madrigal, MSD, Novartis, Novo Nordisk, Pfizer, Roche, Sanofi, Siemens Healthcare GmbH. Research Funding from Gilead Sciences, Boehringer Ingelheim, Nordic Bioscience, Siemens Healthcare GmbH. Speaker Honorarium from MedPublico GmbH, Boehringer Ingelheim. All has been outside of the submitted work. MT has participated in an advisory board for GE Healthcare; and reports speaker’s fee from Echosens, Siemens Healthcare, Tillotts Pharma and Norgine, outside of the submitted work. LV has received a grant and support for travelling and attending meetings from Gilead. He has served as a consultant for Gilead, Pfizer, Astra Zeneca, Novo Nordisk, Intercept pharmaceuticals, Diatech Pharmacogenetics, IONIS, Boehringer Ingelheim and reports being a lecturer or speaker for MSD, Gilead, AlfaSigma, AbbVie, Viatris. In addition, he has participated in advisory boards for Intercept, Pfizer, Gilead, Novo Nordisk. All has been outside of the submitted work. VW-SW served as a consultant or advisory board member for AbbVie, Boehringer Ingelheim, Echosens, Gilead Sciences, Intercept, Inventiva, Merck, Novo Nordisk, Pfizer, ProSciento, Sagimet Biosciences and TARGET PharmaSolutions; and a speaker for Abbott, AbbVie, Echosens, Gilead Sciences and Novo Nordisk. He has received a grant from Gilead Sciences to support fatty liver research and is a co-founder of Illuminatio Medical Technology Limited, outside of the submitted work. YY has received research grants from Biocodex and Gilead, and is a speaker for Novo Nordisk, Gilead, AbbVie, Abdi İbrahim, Bilim İlaç and Echosens, outside of the submitted work. SMF holds a senior clinical investigator fellowship from the Research Foundation Flanders (FWO) (1802154 N). His institution has received grants from Astellas, Falk Pharma, Genfit, Gilead Sciences, GlympsBio, Janssens Pharmaceutica, Inventiva, Merck Sharp & Dome, Pfizer and Roche. He has acted as consultant for AbbVie, Actelion, Aelin Therapeutics, AgoMab, Aligos Therapeutics, Allergan, Astellas, Astra Zeneca, Bayer, Boehringer Ingelheim, Bristoll-Meyers Squibb, CSL Behring, Coherus, Echosens, Eisai, Enyo, Galapagos, Galmed, Genetech, Genfit, Gilead Sciences, Intercept, Inventiva, Janssens Pharmaceutica, Julius Clinical, Madrigal, Medimmune, Merck Sharp & Dome, NGM Bio, Novartis, Novo Nordisk, Promethera and Roche. He has been lecturer for AbbVie, Allergan, Bayer, Eisai, Genfit, Gilead Sciences, Janssens Cilag, Intercept, Inventiva, Merck Sharp & Dome, Novo Nordisk and Promethera, outside of the submitted work. EAT has participated in advisory boards for Intercept, Gilead, Novo-Nordisk and Pfizer, outside of the submitted work. HEM, SAA, and ZMY have nothing to disclose.

Please refer to the accompanying ICMJE disclosure forms for further details.

## References

[1] Ginès P., Graupera I., Lammert F., Angeli P., Caballeria L., Krag A. (2016). Screening for liver fibrosis in the general population: a call for action. Lancet Gastroenterol Hepatol.

[2] Bernstein D., Kovalic A.J. (2022). Noninvasive assessment of fibrosis among patients with nonalcoholic fatty liver disease [NAFLD]. Metabol Open.

[3] Anstee Q.M., Castera L., Loomba R. (2022). Impact of non-invasive biomarkers on hepatology practice: past, present and future. J Hepatol.

[4] Bedossa P. (2014). Utility and appropriateness of the fatty liver inhibition of progression (FLIP) algorithm and steatosis, activity, and fibrosis (SAF) score in the evaluation of biopsies of nonalcoholic fatty liver disease. Hepatology.

[5] Kleiner D.E., Brunt E.M., Van Natta M., Behling C., Contos M.J., Cummings O.W. (2005). Design and validation of a histological scoring system for nonalcoholic fatty liver disease. Hepatology.

[6] Loomba R., Adams L.A. (2020). Advances in non-invasive assessment of hepatic fibrosis. Gut.

[7] Castera L. (2020). Non-invasive tests for liver fibrosis in NAFLD: creating pathways between primary healthcare and liver clinics. Liver Int.

[8] Lazarus J.V., Anstee Q.M., Hagström H., Cusi K., Cortez-Pinto H., Mark H.E. (2021). Defining comprehensive models of care for NAFLD. Nat Rev Gastroenterol Hepatol.

[9] Lazarus J.V., Mark H.E., Anstee Q.M., Arab J.P., Batterham R.L., Castera L. (2022). Advancing the global public health agenda for NAFLD: a consensus statement. Nat Rev Gastroenterol Hepatol.

[10] McPherson S., Hardy T., Dufour J.F., Petta S., Romero-Gomez M., Allison M. (2017). Age as a confounding factor for the accurate non-invasive diagnosis of advanced NAFLD fibrosis. Am J Gastroenterol.

[11] Eslam M., Wong G.L., Hashem A.M., Chan H.L., Nielsen M.J., Leeming D.J. (2021). A sequential algorithm combining ADAPT and liver stiffness can stage metabolic-associated fatty liver disease in hospital-based and primary care patients. Am J Gastroenterol.

[12] Srivastava A., Gailer R., Tanwar S., Trembling P., Parkes J., Rodger A. (2019). Prospective evaluation of a primary care referral pathway for patients with non-alcoholic fatty liver disease. J Hepatol.

[13] Mózes F.E., Lee J.A., Selvaraj E.A., Jayaswal A.N.A., Trauner M., Boursier J. (2022). Diagnostic accuracy of non-invasive tests for advanced fibrosis in patients with NAFLD: an individual patient data meta-analysis. Gut.

[14] Berzigotti A., Tsochatzis E., Boursier J., Castera L., Cazzagon N., Friedrich-Rust M. (2021). EASL Clinical Practice Guidelines on non-invasive tests for evaluation of liver disease severity and prognosis - 2021 update. J Hepatol.

[15] Chalasani N., Younossi Z., Lavine J.E., Charlton M., Cusi K., Rinella M. (2018). The diagnosis and management of nonalcoholic fatty liver disease: practice guidance from the American Association for the Study of Liver Diseases. Hepatology.

[16] Tsochatzis E.A., Newsome P.N. (2018). Non-alcoholic fatty liver disease and the interface between primary and secondary care. Lancet Gastroenterol Hepatol.

[17] Wong V.W.S., Zelber-Sagi S., Cusi K., Carrieri P., Wright E., Crespo J. (2022). Management of NAFLD in primary care settings. Liver Int.

