# Supplementary material for: Real-world evidence on non-invasive tests and associated cut-offs used to assess fibrosis in routine clinical practice
Source: JHEP Rep. 2022 Sep 22;5(1):100596. doi: 10.1016/j.jhepr.2022.100596 (PMC9832273; doi:10.1016/j.jhepr.2022.100596)
Supplement: Multimedia component 1 [file mmc1.pdf]

Real-world evidence on non-invasive tests and associated cut-offs used to assess fibrosis in routine clinical practice

Jeffrey V. Lazarus, Laurent Castera, Henry E. Mark, Alina M. Allen, Leon A. Adams, Quentin M. Anstee, Marco Arrese, Saleh A. Alqahtani, Elisabetta Bugianesi, Massimo Colombo, Kenneth Cusi, Hannes Hagström, Rohit Loomba, Manuel Romero-Gómez, Jörn M. Schattenberg, Maja Thiele, Luca Valenti, Vincent Wai-Sun Wong, Yusuf Yilmaz, Zobair M. Younossi, Sven Francque, Emmanuel A. Tsochatzis

Table of contents

Background..2

Survey...4

Background

EASL International Liver Foundation

The EASL International Liver Foundation (EILF) is a non-profit organisation working globally, leading research, advocacy and education efforts aimed at improving liver health for the greatest number of people. One of EILF's flagship programmes focuses on advancing the policy agenda on non-alcoholic fatty liver disease (NAFLD). Our work covers a broad range of areas, from improving care for affected populations and analysing national policy responses to the delivery of comprehensive public health responses. EILF is currently leading the development of a global NAFLD public health consensus statement with over 200 experts.

Study background

One of the enduring challenges for addressing the burden of NAFLD is ensuring that patients with high degrees of hepatic fibrosis are identified and referred to specialist liver care. Non-invasive tests (NITs) provide a practical way to assess fibrosis risk in patients. NITs fall within two broad categories: 1) scores and ratios based on indirect and/or direct serum biomarkers; and 2) liver stiffness measured by ultrasound or magnetic resonance-based elastography techniques.[1] Currently available NITs are most reliable for ruling out advanced stages of fibrosis (<F3). By applying an upper cut-off to NIT results, the risk of a patient having a pre-defined level of fibrosis (e.g., advanced fibrosis; stage 3-4) can be predicted. The cut-offs employed have important implications for the sensitivity and specificity of the NITs and the size of the indeterminate range, as does the prevalence of the condition in the specific population group. Generally, a low cut-off will improve the sensitivity and negative predictive value, while a high cut-off will improve the specificity and positive predictive value for advanced fibrosis. The negative predictive value of NITs is generally high, meaning that patients with results below the cut-off can be excluded from further investigations with confidence. However, the positive predictive value of the tests is typically lower, meaning that NITs alone are unable to provide a definitive diagnosis.[2]

NITs - used as a stand-alone test or sequentially - are increasingly used in clinical practice, in both primary and secondary care clinics, to identify patients for referral and work-up. In some settings, NITs have facilitated the development of formal care pathways which aim to efficiently and effective link patients to care, especially those with advanced liver disease who require intervention from a hepatologist/liver specialist or multidisciplinary team. In developing these pathways, decisions need to be made about the NIT cut-offs to be used based on the clinical scenario; for example, in primary care where the prevalence of advanced fibrosis is low, a high sensitivity is preferential compared to a specialist care setting where a high specificity is required.

Why this study is needed

While NITs are becoming much more widely utilised as a means of identifying NAFLD patients with advanced fibrosis, little is known about the cut-offs being employed in clinical practice. Many reports include cut-offs for a specific study population, leading to a range of published cut-offs. We hypothesise that the NITs used and the

---

[1] Castera L. Non-invasive tests for liver fibrosis in NAFLD: Creating pathways between primary healthcare and liver clinics. Liver International. 2020 Feb;40:77-81.
[2] Loomba R, Adams LA. Advances in non-invasive assessment of hepatic fibrosis. Gut. 2020 Jul 1;69(7):1343-52.

corresponding cut-offs are widely heterogenous between different healthcare settings and practices. As a first step toward recommending standardised cut-offs, we aim to understand the current practices.

Study aim and sample

To describe the different NITs and corresponding cut-offs being used in routine clinical practice in different health care settings from all regions of the world. This study will leverage a convenience sample of liver health experts participating in the NAFLD consensus statement process.

***Survey* –** please complete the survey and send the completed version to Henry Mark (henry@easl-ilf.org) and Jeffrey Lazarus (jeffrey.lazarus@isglobal.org). If you have questions or encounter difficulties please contact Henry Mark (henry@easl-ilf.org).

Part 1: Basic information

ID	Questions	Answer
1.1	First name	
1.2	Second (family) name	
1.3	Email	
1.4	Phone	
1.5	Specialisation/discipline	
1.6	Main affiliation	
1.7	Clinic/hospital name	
1.8	Clinic setting *(select 1 only)*	☐ Primary Care<br>☐ Secondary care<br>☐ Tertiary care
1.9	Predominant patient population	
1.10	Clinic/department specialisation	
1.11	Clinic city	
1.12	Clinic country	

Part 2: NITs used and cut-offs employed to determine the risk of fibrosis in NAFLD patients in your clinic/department

***For FIB-4 and NAFLD Fibrosis Score, if you use age specific cut-offs please provide these for each age group. If you use age-specific cut-offs for other NITs please indicate this in the target population box**

ID	**Non-invasive test (NITs)**<br><br>***Only check the box if you use this NIT in your clinic***	**Target population** (e.g., general population, diabetic patients, referred patients with)	**Cut-offs**		**Action** (e.g., keep patient in primary care; further assessment needed, refer to hepatology)	**Reference** (e.g., national/international guidelines, clinic guidelines, personal-cut-off) please include URL if available.
2.1	ALT (only) ☐		Low:			
			Int:			
			High:			
2.2	AST/ALT ratio ☐		Low:			
			Int:			
			High:			
2.3	FIB-4 ☐ *	include age range	Low:			
			Int:			
			High:			
		include age range	Low:			
			Int:			
			High:			
		include age range	Low:			
			Int:			
			High:			
		include age range	Low:			
			Int:			
			High:			
2.4	NAFLD Fibrosis Score (NFS) ☐*	include age range	Low:			
			Int:			
			High:			
			Low:			

		include age range	Int:			
			High:			
		include age range	Low:			
			Int:			
			High:			
		include age range	Low:			
			Int:			
			High:			
2.5	AST to platelet ratio Index (APRI) ☐		Low:			
			Int:			
			High:			
2.6	FibroTest® ☐		Low:			
			Int:			
			High:			
2.7	ELF™ score ☐		Low:			
			Int:			
			High:			
2.8	Fibrometer® ☐		Low:			
			Int:			
			High:			
2.9	Hepascore ☐		Low:			
			Int:			
			High:			
2.10	BARD ☐		Low:			
			Int:			
			High:			
2.11	Forns ☐		Low:			
			Int:			
			High:			
2.12	GUCI ☐		Low:			
			Int:			
			High:			

2.13	Transient elastography (FibroScan M probe) ☐	**LSM**	Low:			
			Int:			
			High:			
		**CAP**	Low:			
			Int:			
			High:			
2.14	Transient elastography (FibroScan XL probe) ☐	**LSM**	Low:			
			Int:			
			High:			
		**CAP**	Low:			
			Int:			
			High:			
2.15	Point shear wave elastography (pSWE) ☐ *Please specify manufacturer*:		Low:			
			Int:			
			High:			
2.16	Two-dimensional shear wave elastography (2D-SWE) ☐ *Please specify manufacturer*:		Low:			
			Int:			
			High:			
2.17	Magnetic resonance elastography (MRE) ☐ *Please specify manufacturer*:		Low:			
			Int:			
			High:			
2.18	Other ☐ Please specify:		Low:			
			Int:			
			High:			

Abbreviations: ALT, alanine aminotransferase; AST, aspartate aminotransferase; CAP, controlled attenuation parameter; ELF, Enhanced Liver Fibrosis; FIB-4, Fibrosis-4; FibroTest, fibrosis test; Int, intermediate; LSM, liver stiffness measurement.

Part 3: Formal care pathways and supporting documentation

ID	Questions	Answer	Instructions
3.1	Is there a formal/written **national** risk stratification pathway used by your clinic/hospital?	Yes ☐<br>No ☐	If no skip to 3.4, if yes, please answer 3.2 and 3.2
3.2	Does this national pathway outline the NITs and the cut-offs?		
3.3	Please provide the URL for the national pathway (if available) or email a copy to Henry Mark (henry@easl-ilf.org)		
3.4	Is there a formal/written sub-nation/regional risk stratification pathway use by your clinic/hospital?	Yes ☐<br>No ☐	If no, you have completed the survey. If yes, please answer 3.5 and 3.6
3.5	Does this sub-nation/regional pathway outline the NITs and the cut-offs?		
3.6	Please provide the URL for the sub-nation/regional pathway (if available) or email a copy to Henry Mark (henry@easl-ilf.org)		

Please put any comments or feedback in the box below
